# Supplementary material for: Validation of In Vitro Trained Transcriptomic Radiosensitivity Signatures in Clinical Cohorts
Source: Cancers (Basel). 2023 Jul 5;15(13):3504. doi: 10.3390/cancers15133504 (PMC10340371; doi:10.3390/cancers15133504)
Supplement: Supplementary file 1 [file cancers-15-03504-s001.zip › cancers-2433944-supplementary.pdf]

Supplementary information

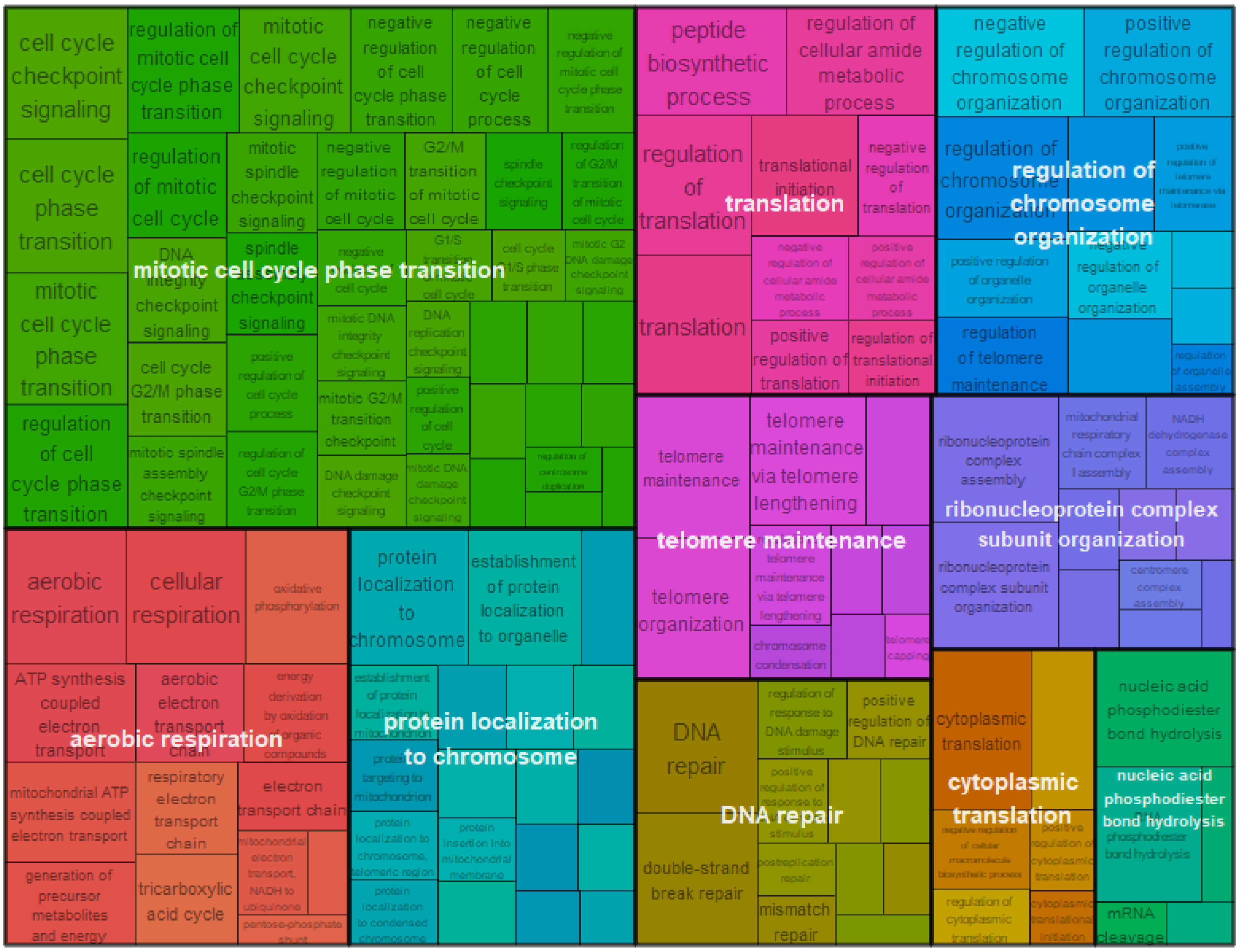

Figure S1. Biological processes identified by GSEA using a list of genes ranked by their difference in univariate hazard ratio between RT and noRT groups with first recurrence as the outcome

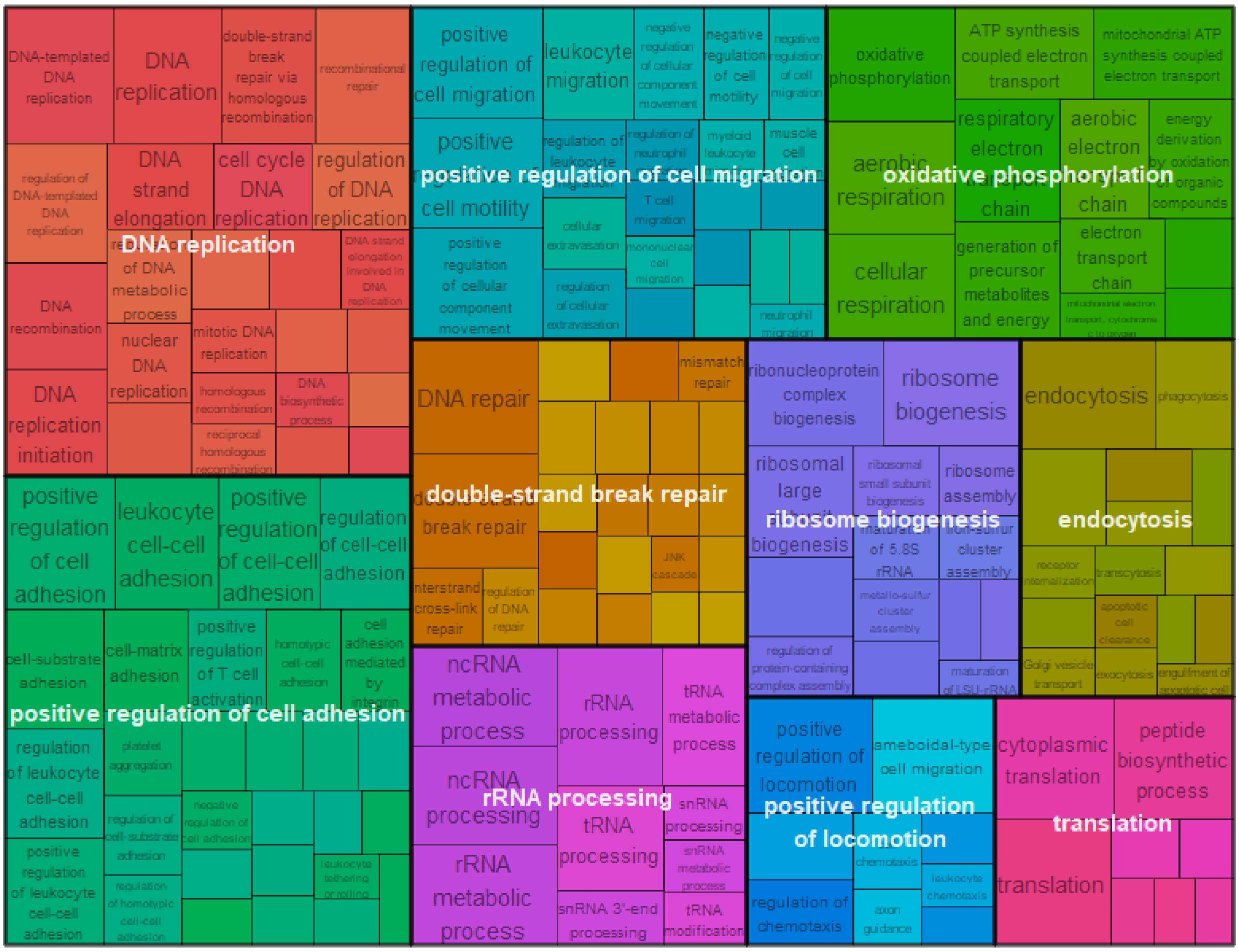

Figure S2. Biological processes identified by GSEA using a list of genes ranked by their difference in univariate hazard ratio between RT and noRT groups with overall survival as the outcome

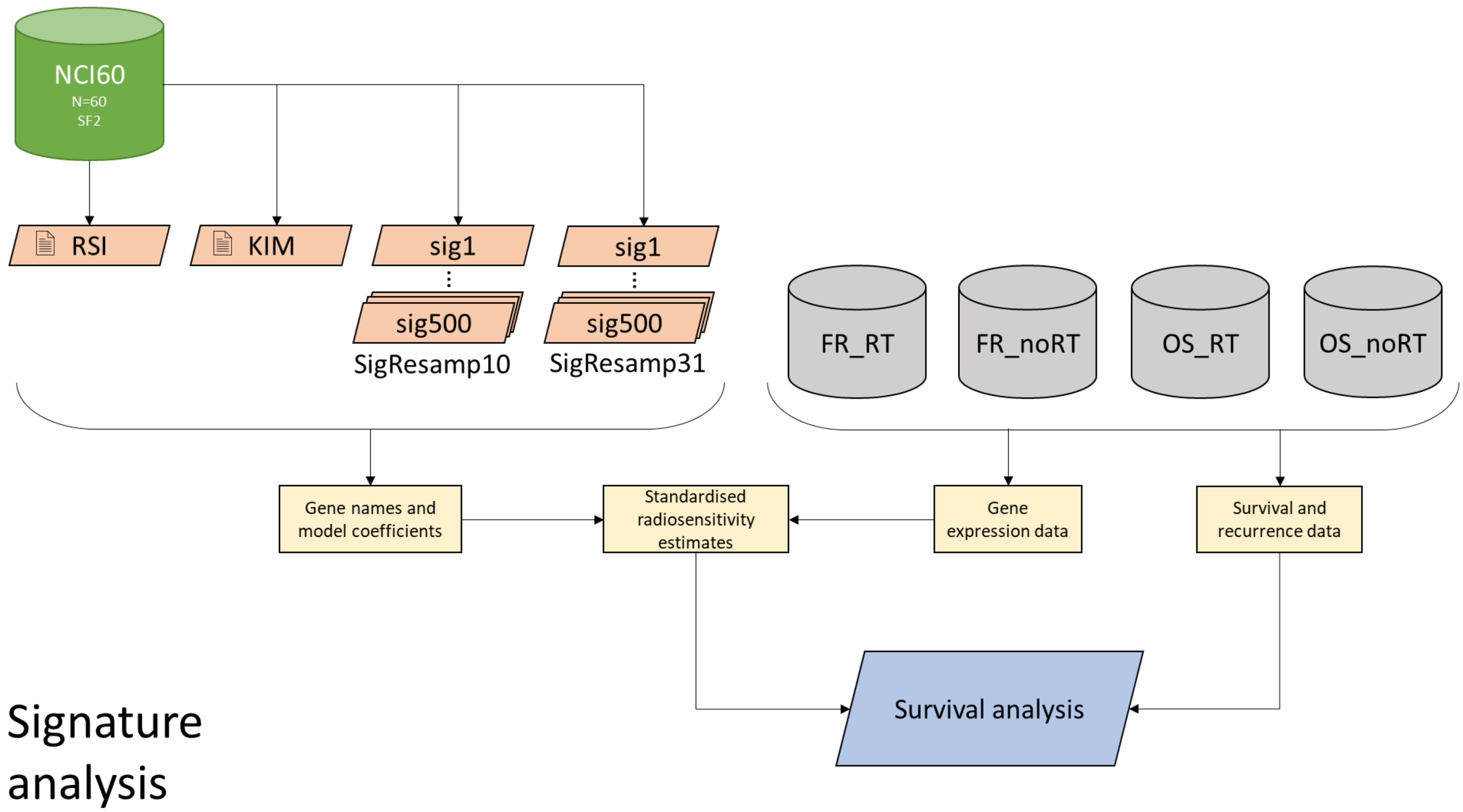

Figure S3. Workflow for assessing the accuracy of published radiosensitivity (RSI and KIM) and resampled signatures (SigResamp10 and SigResamp31) trained on in vitro data and applied in clinical data. Data were pooled over outcome type and RT treatment.

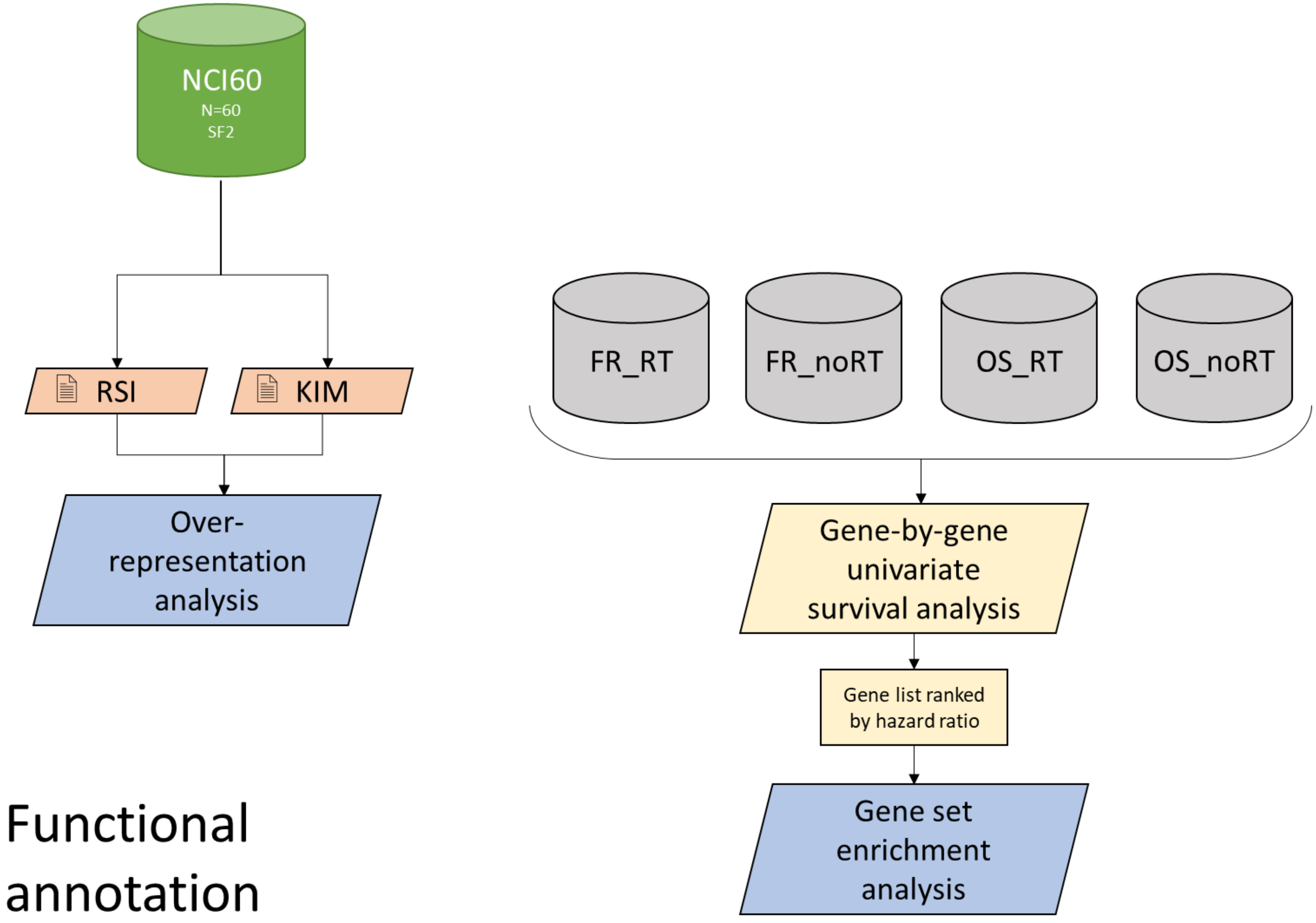

Figure S4. Workflow for overrepresentation analysis of published radiosensitivity (RSI and KIM) and resampled signatures (SigResamp10 and SigResamp31) trained on the NCI60 (left). GSEA using hazard ratios from univariate survival analysis for all genes in clinical data (right).

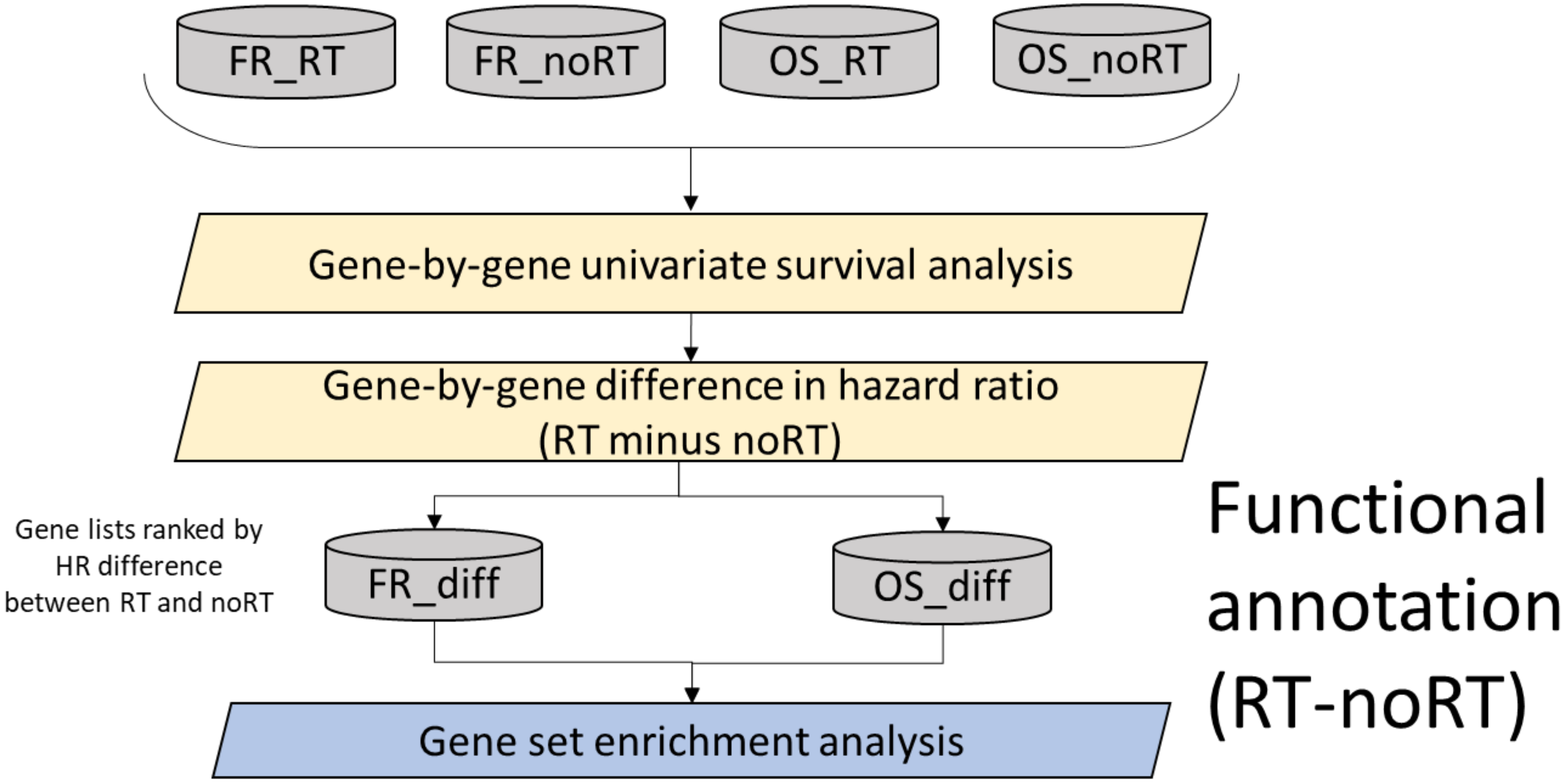

Figure S5. Workflow for GSEA using by gene differences between RT and noRT hazard ratios from univariate survival analysis in clinical data.

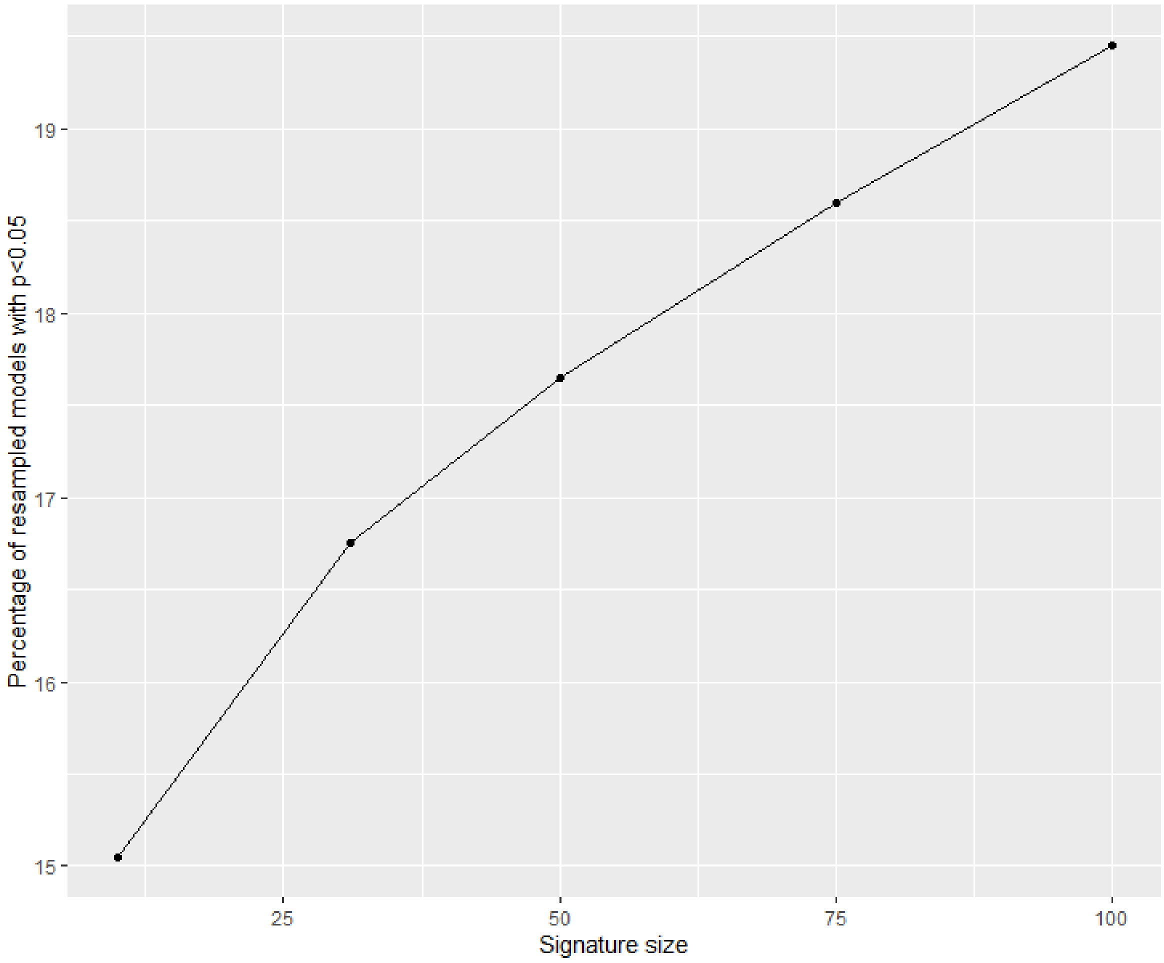

Figure S6. Percentage of resampled signature models with a model p-value of <0.05 plotted for signature sizes of 10, 31, 50, 75 & 100.

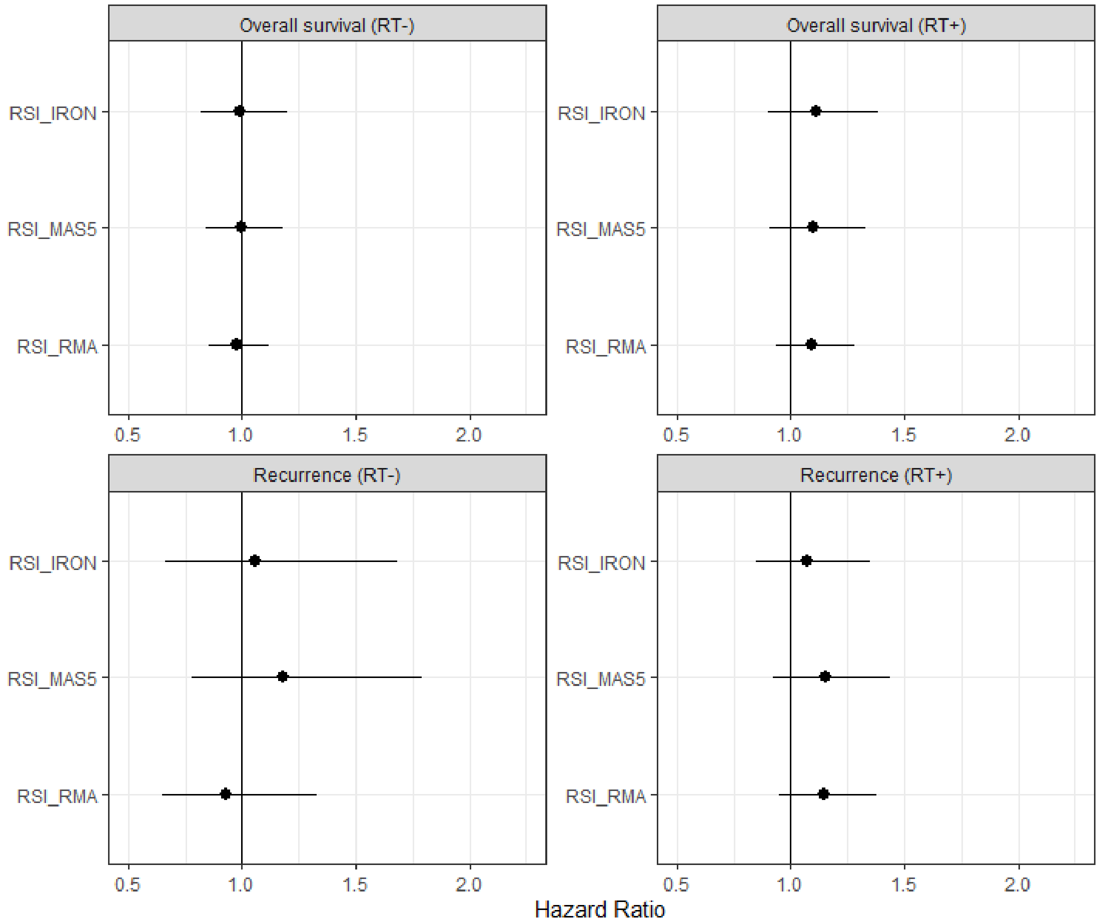

Figure S7. Hazard ratios for RSI signature using different normalisation algorithms. The largest difference was in the recurrence group without radiation treatment where using RMA estimated a HR of 0.93 (95%CI: 0.65–1.33) while the same estimate using MAS5 was 1.18 (95%CI: 0.78–1.80).
